# Improved functional outcomes in a patient with SLE longitudinal extensive transverse myelitis: A case report

**DOI:** 10.1097/MD.0000000000046534

**Published:** 2025-12-26

**Authors:** Lama Quraiba, Dillon Welch, Ryan Welch, Katherine Weir, Dean Wundrach, Sarah Mahasin, Steven Jackson

**Affiliations:** a College of Medicine, Alfaisal University, Riyadh, Saudi Arabia; b University of Minnesota Medical School, Minneapolis, Minnesota; c Chicago College of Osteopathic Medicine, Downers Grove, IL; d University of Minnesota Medical School, Minneapolis, MN; e Health Partners/Regions Hospital, Saint Paul, MN .

**Keywords:** case report, myelitis, rehabilitation, spinal cord injuries, systemic lupus erythematosus

## Abstract

**Rationale:**

Longitudinal extensive transverse myelitis (LETM) is a rare form of acute transverse myelitis occurring in 1%–2% of systemic lupus erythematosus (SLE) cases. It often leads to severe sensory and motor deficits with bowel and bladder dysfunction. Reports of complete functional recovery in SLE-related LETM are limited, and the significance of early and aggressive intervention remains insufficiently understood.

**Patient concerns:**

A 31-year-old female with prediabetes and iron deficiency anemia was hospitalized for hematochezia and diarrhea. On the third day of admission, she experienced sudden bilateral lower extremity weakness resulting in a fall.

**Diagnoses:**

Further evaluation revealed findings consistent with SLE-related LETM, confirmed through clinical, laboratory, and imaging assessments.

**Interventions:**

The patient received aggressive medical management for SLE-LETM, followed by a multidisciplinary rehabilitation program. Over 1 year, she completed acute inpatient rehabilitation and continued outpatient physical, occupational, and aquatic therapies. She also used an ankle-foot orthosis, a cane, and a front-wheeled walker as needed during her recovery.

**Outcomes:**

Over the course of 1 year, the patient demonstrated marked progressive improvement, ultimately achieving functional ambulation with assistive devices and a Berg Balance Scale score of 48/56.

**Lessons:**

This case underscores the importance of prompt diagnosis, aggressive medical therapy, and early multidisciplinary rehabilitation in optimizing recovery in SLE-LETM. Although this condition is often associated with poor outcomes, significant functional recovery is achievable with timely and intensive management. Further studies are needed to clarify the role of early rehabilitation in long-term outcomes.

## 1. Introduction

Acute transverse myelitis (ATM) is a rare inflammatory disease of the spinal cord. The annual incidence ranges from 1 to 4 cases per million.^[1]^ Longitudinal extensive transverse myelitis (LETM) is a rare type of ATM that involves inflammation of more than 3 continuous spinal segments.^[2]^ ATM occurs in 1% to 2% of SLE patients, and the LETM variant is even more rarely seen and more severe than ATM.^[2]^ A positive association between SLE and LETM has been reported in the literature.^[3]^ LETM commonly presents with fast and progressive dysfunction of the nervous system including sensory loss, motor loss, and incomplete bladder control.^[2]^ Here we describe a case of a young female with LETM as the first manifestation of SLE who achieved complete functional recovery with extensive rehabilitation.

## 2. Clinical presentation

**Figure 1.**
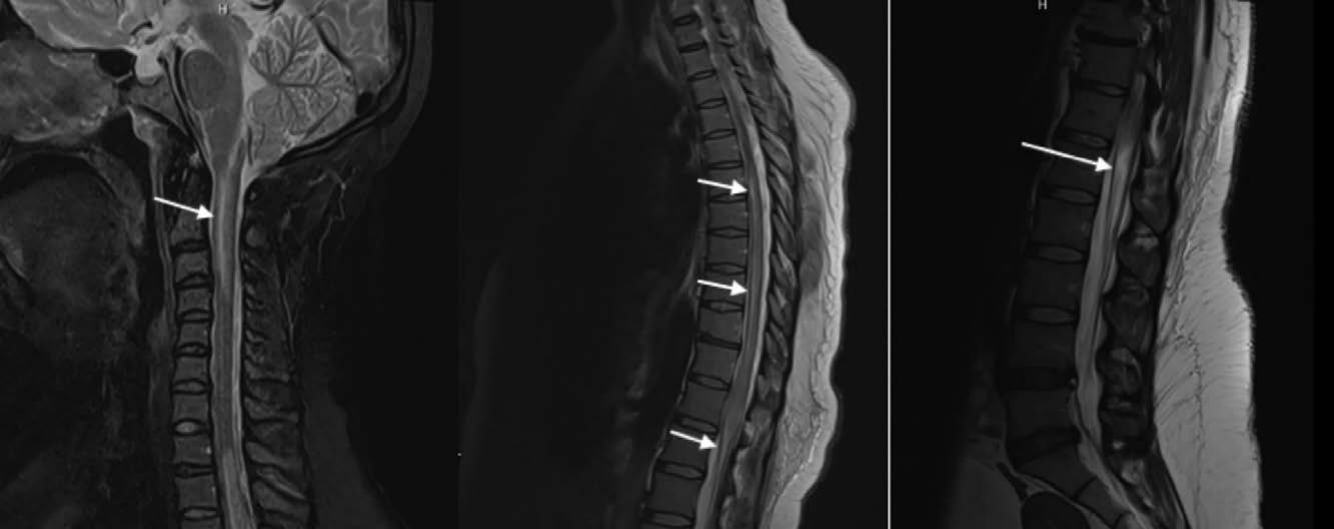
T2 MRI hyperintensity extending from the right cervical medullary junction to C4-5 interspace (image 1), and patchy enhancement extending from T5-6 to conus medullaris (images 2 and 3). MRI = magnetic resonance imaging.

A 31-year-old female with prediabetes and iron deficiency anemia presented to the emergency room with a 3-week history of vomiting, nausea, hematochezia, and diarrhea (Table 1). The review of the systems was positive for arthralgia, uveitis, anorexia, weight loss, and night sweats. The patient was febrile (T 103 F) and tachycardic (HR 110) (Table 1). On initial physical examination, both abdominal and neurologic examination were unremarkable. She was admitted to the hospital for possible inflammatory bowel disease. After receiving an esophagogastroduodenoscopy, the patient was diagnosed with candida esophagitis and chronic active gastritis (Table 1). On the third day of this patient’s admission, she suffered a fall and was unable to get up. Subsequent examination demonstrated significant bilateral lower extremity weakness along with urinary retention requiring catheterization, which showed a post-void residual volume >700 cc (Table 1). Her neurologic examination showed intact cranial nerves, bilateral upper extremity strength of 5/5, and bilateral lower extremity strength of 1/5 at the hip and 0/5 distally. Her sensation was intact, although she had numbness in a saddle-shape distribution and her bilateral lower extremities were areflexic. The patient received a lumbar puncture and the cerebrospinal fluid analysis showed elevated white blood cell levels of 173 and protein levels of 157 (Table 1). The infection panel was negative. MRI showed spinal cord hyperintensity extending from the right cervical medullary junction to the C4-5 interspace. There was also an extensive central cord signal abnormality, slight expansion, and patchy enhancement extending from T5-6 to the conus medullaris (Table 1). The MRI did not note any central canal or foraminal stenosis. There was also no enhancement of the cauda equina. These MRI findings are consistent with ATM (Fig. 1). Rheumatologic markers were also ordered and showed: positive antinuclear antibody (ANA) > 1:12, ds-DNA 46.2, Antismith 26, Anti-U1-RNP 104, low complement C3/C4 32, 3.6. These results were consistent with a new diagnosis of systemic lupus erythematosus with > 10 points on the 2019 EULAR/ACR classification. She was diagnosed with SLE longitudinal extensive transverse myelitis and was subsequently started on aggressive medical therapy including high-dose prednisone with a slow taper starting at 60 mg intravenous (IV) daily, plasmapheresis, weekly Rituximab for 1 month, biweekly Cyclophosphamide for 1 month, and Hydroxychloroquine (Table 1). The patient remained hospitalized for 20 days before she was transferred to the acute inpatient rehabilitation floor.

**Table 1:** Timeline of clinical course for SLE–LETM case.

Time point	Event	Key details/findings
3 wk before admission	Symptom onset	Nausea, vomiting, hematochezia, diarrhea, arthralgia, uveitis, weight loss, night sweats
Day 0 (hospital admission)	Hospitalization for gastrointestinal symptoms	Febrile (103°F), tachycardic (HR 110); suspected inflammatory bowel disease
Day 1–2	Diagnostic workup	EGD: candida esophagitis and chronic active gastritis
Day 3	Neurologic event	Sudden bilateral lower extremity weakness, urinary retention (>700 cc post-void residual)
Day 3–5	Neurologic evaluation	MRI: cord hyperintensity from medulla to conus; CSF: elevated WBC (173) and protein (157)
Day 6–7	Diagnosis	SLE-LETM confirmed (positive ANA, dsDNA, anti-Smith, anti-U1-RNP; low C3/C4)
Day 7–27	Acute medical treatment (20 d)	High-dose IV prednisone (60 mg daily, taper), plasmapheresis, rituximab, cyclophosphamide, hydroxychloroquine
Day 27–48	Acute inpatient rehabilitation (21 d)	ASIA C (T10); intensive PT/OT with FES, strengthening, and ADL training; improved LE strength
At discharge (~day 48)	Functional gains	Ambulated 30 ft with AFOs and walker (CG assist), Berg score 32/56; modified independent in ADLs
6 mo post-discharge	Continued outpatient rehab	Participated in physical, occupational, and aquatic therapy
1-year follow-up	Functional recovery	Ambulating with cane/FWW; Berg score 48/56; LE strength improved; partial bladder control regained

ANA = antinuclear antibody, anti-Smith = anti-Smith antibody, anti-U1-RNP = anti-U1 ribonucleoprotein antibody, CSF = cerebrospinal fluid, dsDNA = double-stranded DNA, HR = heart rate, EGD = esophagogastroduodenoscopy, MRI = magnetic resonance imaging, SLE-LETM = systemic lupus erythematosus-related longitudinal extensive transverse myelitis.

### 2.1. Acute inpatient rehabilitation

Evaluation upon admission to the inpatient rehabilitation site noted bilateral lower limb weakness (Tables 1and 2). Initial physical therapist assessment reported maximal assistance needed for bed-chair transfers and dependent toilet transfers. The initial occupational therapist assessment revealed assistance for lower body dressing and bathing. Medically, the patient also suffered from a neurogenic bowel and bladder with urinary incontinence. The American Spinal Cord Injury Association (ASIA) impairment scale on admission was T10 (ASIA C). The occupational therapy program focused on bilateral upper extremity strengthening using an arm bike, weights, and resistance exercises. The physical therapy program included 20 minutes a day on a functional electrical stimulation cycle which bilaterally targeted the erector spinae, gluteals, hamstrings, quadriceps, tibialis anterior, and gastrocnemius muscles. Additionally, massage therapy and acupuncture were done. She spent 21 days total in acute inpatient rehabilitation before discharging home.

### 2.2. At discharge

The patient’s weakness had improved and physical examination findings at that time showed bilateral improvement in lower extremity weakness (Tables 1 and 2). She was able to tolerate an active time of 19:36 minutes out of 22:21 on a functional electrical stimulation bike with a total distance cycled of 2.32 miles. She was able to stand with ankle-foot orthoses, shoes, and a wide walker. With close contact guard assist, she was able to ambulate 30 ft on the parallel bars. In addition, her Berg balance test score was 32/56. Occupational therapist evaluation showed the patient was able to perform activities of daily living in a wheelchair (graded as modified independent). She could use a slide board, transfer to the bathroom, eat, shower, dress, and toilet independently. The patient remained on a bowel and bladder program, but she regained some bladder control. She still relied on self-intermittent catheterization every 4 hours and was prescribed Oxybutynin to manage her urinary incontinence. She was also prescribed Gabapentin 600 mg 3 times a day to manage neuropathic pain.

**Table 2:** Bilateral lower muscle strength throughout the treatment course.

	Diagnosis		Discharge		1-yr follow up	
	Right	Left	Right	Left	Right	Left
Hip flexion	1/5	1/5	3/5	2/5	4/5	4/5
Knee flexion	N/A	N/A	3/5	3/5	5/5	5/5
Knee extension	0/5	1/5	3/5	3/5	5/5	5/5
Dorsiflexion	N/A	N/A	4/5	3/5	5/5	5/5
Plantar Flexion	0/5	0/5	4/5	4/5	5/5	5/5

N/A = not applicable.

### 2.3. At the 1-year follow-up

The patient was ambulating using a cane or front-wheeled walker to ambulate short distances and a wheelchair for longer distances. Remarkably, her Berg balance score was 48/56. She could ambulate 20 feet in 13 seconds with a front-wheeled walker and 20 feet in 35 seconds with a cane. She also completed aquatic therapy and reported notable improvements in her strength (Table 1). At 1 year, her physical examination showed significant improvement in her lower extremity power bilaterally (Tables 1 and 2).

## 3. Discussion

Myelopathy is considered one of the 19 neuropsychiatric diseases associated with SLE by the American Rheumatology Society.^[3]^ SLE myelitis, a form of myelopathy, can be further classified based on the spinal cord involvement into transverse (SLE-TM) or longitudinal extensive myelitis (SLE-LETM), with LETM spinal lesions spanning more vertebrae (at least 3 consecutive).^[2]^ The pathophysiology of this disease is not well understood, with current hypotheses suggesting the etiology stems from vasculitis of the spinal cord.^[4]^

Treatment recommendations for SLE myelitis are based on case studies and trials that treated TM secondary to etiologies other than SLE; therefore, the evidence is limited. However, the available evidence has suggested that high-dose corticosteroids combined with immunosuppressants such as Cyclophosphamide can help improve long-term functional outcomes of SLE myelitis.^[2,3]^ Rituximab and plasmapheresis have also been recommended to treat severe and acute-onset SLE myelitis.^[5]^ Furthermore, early recognition of the disease and initiation of treatment within 2 weeks of symptom onset has been associated with better functional outcomes.^[6]^ In this case, the patient’s medical management aligns with the current standard of treatment with high-dose prednisone and cyclophosphamide, as well as plasmapheresis, Rituximab, and Hydroxychloroquine. In addition to medical management, this patient received intensive acute inpatient rehabilitation including physical therapy, occupational therapy, massage therapy, and acupuncture, with continued outpatient rehabilitation and aquatic therapy. There are no established guidelines for rehabilitation therapies for recovery from SLE myelitis.

The literature notes that the functional recovery of SLE-LETM patients is poor.^[2,3]^ In one study that examined 22 patients with SLE-LETM, 86% had some sort of remaining disability from the disease after treatment. These residual disabilities ranged from mild to severe with deficits in sensation, motor movement, and/or bladder control.^[7]^ Although rare, complete functional recovery has been observed. Jackson et al reports a similar case in which a woman with SLE- LETM made a functional recovery from the classification of ASIA A initially to AIS D after treatment with aggressive medical management in combination with intensive rehabilitation.^[8]^

Many studies have attempted to identify prognostic factors for SLE myelitis. Severe initial neurologic deficits have been associated with worse long-term prognosis.^[5,6,9]^ Wang et al performed a systematic review of prognostic factors for SLE myelitis and found that patients with AIS scores of A-C on initial presentation are more likely to have a poor prognosis.^[5]^ More extensive spinal cord lesions and urinary sphincter dysfunction have also independently been associated with worse neurologic outcomes.^[10]^ Furthermore, multiple studies have linked low glucose in the cerebrospinal fluid (CSF), also known as hypoglycorrhachia, to unfavorable neurologic outcomes for patients with SLE myelitis.^[5,9]^ It has been proposed that hypoglycorrhachia in SLE myelitis could result from increased glucose uptake by inflammatory cells that infiltrate the spinal cord, and thus, it may be a marker of central nervous system inflammation.^[5]^ Although it is not documented whether the patient in this case had hypoglycorrhachia, she made a full functional recovery despite an (AIS C) score on presentation with urinary incontinence.

Limitations of this case report include the lack of documentation of the patient’s outpatient rehabilitation course. Her inpatient rehabilitation course and therapies are adequately reported but after discharge, the rehabilitative information is scarce. It is known that she continued to attend therapies outpatient, but her course regimen and progress are not tracked until her 1- year follow-up appointment. Another limitation is the lack of full information of potential prognostic factors, including glucose count in the CSF.

## 4. Conclusion

This case demonstrates how a rehabilitation treatment course involving occupational therapy, physical therapy, aquatic therapy, massage therapy, and acupuncture, in conjunction with medical management, succeeded in achieving full functional recovery from SLE-LETM. SLE-LETM often leaves patients with long-term deficits, but functional recovery is possible with an early and intensive treatment plan. Although prognostic factors that influence functional outcomes for patients should be considered, the implementation of comprehensive rehabilitation therapies could play an important role in the functional recovery of SLE-LETM. More research is needed to determine the true impact of rehabilitation on SLE-LETM recovery and the optimal recommendations for the rehabilitation course.

## Author contributions

**Conceptualization:** Lama Quraiba, Dillon Welch, Katherine Weir, Dean Wundrach, Sarah Mahasin.

**Investigation:** Steven Jackson.

**Resources:** Lama Quraiba, Dillon Welch, Katherine Weir, Dean Wundrach, Sarah Mahasin, Steven Jackson.

**Supervision:** Sarah Mahasin, Steven Jackson.

**Writing – original draft:** Lama Quraiba, Dillon Welch, Ryan Welch, Katherine Weir, Dean Wundrach.

**Writing – review & editing:** Lama Quraiba, Dillon Welch, Ryan Welch, Sarah Mahasin.
